# Racial-ethnic disparities in psychological distress during the COVID-19 pandemic in the United States: the role of experienced discrimination and perceived racial bias

**DOI:** 10.1186/s12889-023-15912-4

**Published:** 2023-05-25

**Authors:** Ming Wen, Lu Shi, Donglan Zhang, Yan Li, Zhuo Chen, Baojiang Chen, Liwei Chen, Lu Zhang, Hongmei Li, Jian Li, Xuesong Han, Dejun Su

**Affiliations:** 1 Faculty of Social Sciences, University of Hong Kong, Pok Fu Lam, Hong Kong; 2 Department of Sociology, University of Utah, Salt Lake City, UT 84112 USA; 3 Department of Public Health Sciences, Clemson University, Clemson, SC 29634 USA; 4 Division of Health Services Research, Department of Foundations of Medicine, New York University Long Island School of Medicine, Mineola, NY 11501 USA; 5 Department of Population Health Science and Policy, Icahn School of Medicine at Mount Sinai, New York, NY 10029 USA; 6 Department of Health Policy and Management, College of Public Health, University of Georgia, Athens, GA 30602 USA; 7 Department of Biostatistics and Data Science, School of Public Health, University of Texas Health Science Center at Houston, 1616 Guadalupe, Suite 6.300, Austin, TX 78701 USA; 8 Department of Epidemiology, Fielding School of Public Health, University of California Los Angeles, Los Angeles, CA 90095 USA; 9 Department of Media, Journalism and Film, Miami University, Oxford, OH 45056 USA; 10 Department of Environmental Health Sciences, Fielding School of Public Health, School of Nursing, University of California Los Angeles, Los Angeles, CA 90095 USA; 11 Surveillance and Health Equity Science, American Cancer Society, Atlanta, GA 30303 USA; 12 Department of Health Promotion, College of Public Health, University of Nebraska Medical Center, Omaha, NE 68198 USA

**Keywords:** Psychological distress, Racial-ethnic disparities, COVID-19, Experienced discrimination, Perceived racial Bias

## Abstract

**Background:**

Research on mental health disparities by race-ethnicity in the United States (US) during COVID-19 is limited and has generated mixed results. Few studies have included Asian Americans as a whole or by subgroups in the analysis.

**Methods:**

Data came from the 2020 Health, Ethnicity, and Pandemic Study, based on a nationally representative sample of 2,709 community-dwelling adults in the US with minorities oversampled. The outcome was psychological distress. The exposure variable was race-ethnicity, including four major racial-ethnic groups and several Asian ethnic subgroups in the US. The mediators included experienced discrimination and perceived racial bias toward one’s racial-ethnic group. Weighted linear regressions and mediation analyses were performed.

**Results:**

Among the four major racial-ethnic groups, Hispanics (22%) had the highest prevalence of severe distress, followed by Asians (18%) and Blacks (16%), with Whites (14%) having the lowest prevalence. Hispanics’ poorer mental health was largely due to their socioeconomic disadvantages. Within Asians, Southeast Asians (29%), Koreans (27%), and South Asians (22%) exhibited the highest prevalence of severe distress. Their worse mental health was mainly mediated by experienced discrimination and perceived racial bias.

**Conclusions:**

Purposefully tackling racial prejudice and discrimination is necessary to alleviate the disproportionate psychological distress burden in racial-ethnic minority groups.

## Introduction

Mental health disorders were a significant public health burden even before COVID-19 [1]. The patterns of mental health disparities by race-ethnicity were complex, often depending on the specific outcomes and covariates controlled in the analysis [2–4]. While some national survey data showed that Black and Hispanic individuals were more likely than White individuals to meet depressive disorder criteria [5], other studies suggested that mental health disorder rates were either similar or lower in Black, Hispanic, and Asian individuals compared with White individuals [6, 7].

During the COVID-19 pandemic, mental health has worsened in the United States (US) [8–11]. National data showed that, in April 2020, 13.6% of US adults reported symptoms of severe psychological distress, relative to 3.9% in 2018 [12]. Depressive symptoms also became considerably more prevalent during COVID-19 compared with before [13] and cumulative evidence from a rapidly growing literature pointed to a disproportionate burden of mental health among racial-ethnic minorities [14–21] .

An important driver of the disproportionate mental health burden among racial-ethnic minorities during the pandemic include heightened racism and xenophobia and socioeconomic disadvantages among certain minority groups. For example, Asians of all ethnicities, scapegoated during the disastrous COVID-19 global pandemic, experienced a striking rise in anti-Asian discrimination in various forms, including but not limited to verbal assaults and physical attacks [22–25]. Despite this disturbing trend, there were substantial variations in the likelihood of reporting personal experience of discrimination across subgroups of Asian Americans. Evidence from a 2021 national survey suggested that Chinese were more likely to report experience of discrimination than Koreans, Filipinos, and South Asians after adjusting for demographics, socioeconomic status, and other variables [26].

The pandemic has also ramped up racism against non-Asian racial-ethnic minority groups [27–29]. Findings from analyzing nationally representative survey data with an embedded vignette experiment about roommate selection indicate that COVID-19 has fueled prejudice and discrimination against East Asian, South Asian, and Hispanic hypothetical room-seekers partly because of their perceived lack of responsibility and/or cultural compatibility [30]. A Pew study conducted in April 2021 revealed that 41% of Black participants reported seeing people acting uncomfortable around them, followed by 27% among Asian and Hispanic participants, compared with 17% among White participants [31]. Recent evidence also showed that limited English proficiency was a strong predictor of experienced racial-ethnic discrimination [32], indicating alarming xenophobic sentiments and behaviors against people perceived as less “American,” including both Asian and non-Asian populations [30].

At the same time, COVID-19 has augmented socioeconomic disadvantages among certain minority groups. Black and Hispanic Americans, on the whole, and some Asian subgroups bear the brunt of the economic crisis sparked by the pandemic. Data from a survey conducted in August-September 2021 showed that more than half of Hispanic (57%) and Black (56%) participants in the US reported facing serious financial problems in the previous few months, as did 32% of Asian households and 29% of White households [33]. While Asian Americans, as an aggregate group, do well on measures of economic well-being compared with other racial-ethnic groups [34], they also are the most socioeconomically diverse, having the highest income inequality of any racial-ethnic group in the US: the median household incomes among East Asian and South Asian groups are higher than those among Southeast Asian groups and higher than the national average [35].

Due to COVID-19 triggered societal changes [6], the landscape of mental health disparities by race-ethnicity in the US may have changed. It is conceivable that the increased pandemic-induced prejudice against Asians and the disproportionately worsened financial/job hardship in some minority groups are likely transferring to their heightened stress and distress, thereby harming their mental well-being. Yet, evidence is limited and mixed as to racial-ethnic disparities in mental health during the pandemic. On the one hand, findings from national surveys showed that Hispanic respondents were more likely to report an increase in psychological distress than White and Black respondents after the pandemic began [10, 12, 36]. Among Asian Americans, while they exhibited a lower prevalence of mental disorders than other major racial-ethnic groups before the pandemic [6], as of May 2020, there had been a sevenfold increase in depression and anxiety prevalence compared to 2019 [37]. On the other hand, several studies found no evidence of worse mental health among racial-ethnic minority respondents compared to non-Hispanic White respondents [13, 38]. Few studies included Asians in these group comparisons.

Leveraging data from a nationally representative survey, the current study has two main purposes. First, we performed mental health comparisons across four major racial-ethnic groups in the US, including White, Black, Hispanic, and Asian populations, and examined Asian subgroup variations. Second, we explored the mediating role of experienced discrimination and perceived racial bias in contributing to mental health disparities by race-ethnicity after socioeconomic status (SES) is controlled. A voluminous literature has routinely documented the positive SES-health relationship [39] and confirmed that much of racial/ethnic health disparities are attributable to SES [40]. What remains less unknown is the role of factors beyond SES such as experienced or perceived racism in contributing to mental health differences across different racial/ethnic minorities. We hypothesized that racial-ethnic minority respondents would report greater psychological distress than non-Hispanic White respondents due to the COVID-19-fueled xenophobia. We also expected that these group differences would be largely attributable to experienced discrimination and perceived racism, independently of socioeconomic impacts.

## Methods

### Participants and data collection

Data came from the 2020 Health, Ethnicity, and Pandemic Study (HEAP), based on a nationally representative sample of 2,709 community-dwelling adults aged 18 and over in the US. The survey was web-based, conducted in both English and Spanish, and fielded by the National Opinion Research Center (NORC) at the University of Chicago in October 2020. We oversampled minority groups, including Asian Americans and Pacific Islanders, Hispanics, and Blacks. Detailed description of the survey is available elsewhere [41]. The NORC Institutional Review Board reviewed and approved the survey. Informed consent was obtained from all participants.

### Measures

The outcome of interest in this study was psychological distress, measured by the Kessler Distress Scale–6 (K6), a well-validated 6-item inventory rated on a 5-point Likert-type scale [42]. The K6 items assessed the frequency of non-specific psychological distress symptoms, including feeling “nervous,” “hopeless,” “restless or fidgety,” “depressed,” “everything was an effort,” and “worthless,” in the past 30 days. The responses included five options, ranging from “none of the time” (coded zero) to “all the time” (coded four). The six items were summed to yield a number between zero and 24, with a score of 13 or higher indicating severe distress [43], and a score between 5 and 12 indicating moderate distress [44]. In this sample, the K6 scale exhibited excellent internal consistency and reliability with a Cronbach’s alpha value of 0.91.

Self-reported race-ethnicity was categorized as non-Hispanic White; Hispanic, any race; non-Hispanic Black; non-Hispanic Asian; and non-Hispanic Other (including multiracial). The Asian background was further disaggregated into three subgroups: East Asian (Chinese, Japanese, and Korean), South Asian (Asian Indian, Bangladeshi, Nepalese, and Pakistani), and Southeast Asian (Burmese, Cambodian, Filipino, Hmong or Miao, Indonesian, and Vietnamese) subgroups. The three East Asian ethnic subgroups were further separately analyzed.

Prejudice and discrimination were measured by two variables. Experienced discrimination was assessed by dichotomous responses to the question, “Have you personally experienced any discrimination or unfair treatment because of your racial or ethnic background during the COVID-19 pandemic?” Perceived bias toward one’s race-ethnicity was captured by a scale consisting of eight items based on statement such as “I believe the country has become more dangerous for people in my racial-ethnic group because of the Coronavirus” and “People of my race-ethnicity are more likely to lose their job because of the Coronavirus” [14]. The response options ranged from “strongly disagree” (coded as 1) to “strongly agree” (coded as 4). The scale exhibited excellent internal consistency and reliability with a Cronbach’s alpha value of 0.89.

Socioeconomic resources were captured by four variables: educational attainment (five levels), household income (18 levels), job changed for worse during the pandemic (coded as 1 if the respondent experienced at least one of the following events: lost jobs, closed personal business permanently or temporarily, furloughed, and experienced a pay cut or reduction of business income), and having health insurance (dichotomous) at the survey time. Other covariates included age group (18–29, 30–44, 45–59, 60+), gender (male vs. female), marital status (married/cohabitating vs. unmarried and not cohabitating), number of children in the household (capped at 10), and immigrant status (US-born vs. foreign-born).

### Statistical analysis

Those who had missing values in race-ethnicity (*n* = 5), the primary exposure, or psychological distress (*n* = 38), the outcome, were excluded (*n* = 43). Missing values in other independent variables were imputed by the predicted values of regression of the imputed variable on psychological distress, age group, gender, race-ethnicity, marital status, number of children, education, and household income [45]. The analytic sample comprised White, Black, Hispanic, Asian, and Other respondents (*n* = 2,666).

Next, weighted sample statistics were generated. Prevalence rates of severe and moderate psychological distress in the whole sample and by race-ethnicity were computed and graphed.

To test whether the observed group differences were significant, six chi-square tests were performed, corresponding to six contingency tables constructed between moderate or severe psychological distress (dichotomous) and group membership defined by (1) five main racial-ethnic groups (i.e., White, Black, Latino, Asian, Other respondents), (2) four major Asian subgroups (i.e., East-Asian, South-Asian, Southeast Asian, and Other Asian respondents), and (3) six more detailed Asian subgroups (i.e., Chinese, Japanese, Korean, South-Asian, Southeast Asian, and Other Asian respondents).

Associations of race-ethnicity with psychological distress (continuously measured) were estimated using weighted multivariable linear regression and the results were expressed as point estimate (β) with standard errors also provided. Two-sided hypothesis testing was conducted at the significance level α = 0.05. Multivariable models were calculated in three steps: Model I adjusted for age, gender, marital status, number of children, and immigrant status; Model II added socioeconomic status (SES) variables to Model I, including education, household income, job change, and health insurance; and Model III added the two discrimination variables to Model II. No alarming collinearity in the regression analysis was detected; the correlation coefficient between the two discrimination variables, experienced discrimination and perceived racial bias, was 0.33, the largest among all the correlation coefficients among the covariates.

A mediation analysis was then carried out to investigate the role of experienced and perceived racial bias (mediators) in the overall effect of race-ethnicity on psychological distress. Following a widely applied approach to estimating regression models with multiple mediators [30, 46], simultaneous equations in a seemingly unrelated regression framework were estimated with the standard error corrected using bootstrapping and bias-corrected confidence intervals. Sensitivity analyses were conducted with weighted logistic regression and found no qualitative differences in regression results with or without missing value imputation.

## Results

**Fig. 1:**
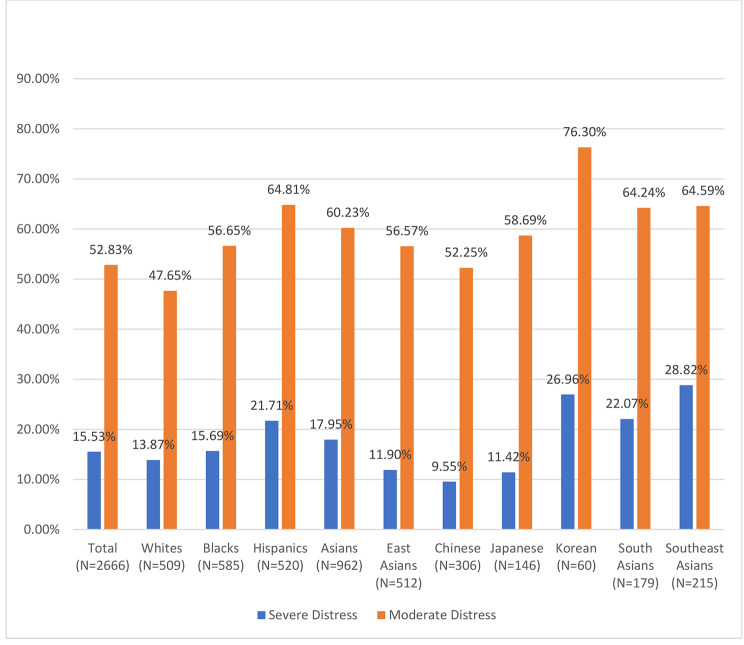
Prevalence of Severe and Moderate Psychological Distress by Race-ethnicity

Figure 1 shows the distribution of psychological distress. A comparison across the four major racial-ethnic groups in the US revealed that Hispanic respondents had the highest prevalence of severe (22%) and moderate (65%) psychological distress, followed by Asian (18%; 60%) and then Black (16%; 57%) respondents, with non-Hispanic White respondents exhibiting the lowest prevalence of severe (14%) and moderate (48%) psychological distress. Among Asian respondents, East Asians had lower prevalence of psychological distress (12%; 57%) than South Asians (22%; 64%) and Southeast Asians (29%; 65%). However, Koreans had the highest prevalence of moderate distress (76%) and the second highest prevalence of severe distress (27%) among all the racial-ethnic groups included in this figure. The observed group differences were all statistically significant at the 5% level based on the results from the series of chi-square tests.

Table 1 presents weighted statistics in the whole sample and in specific racial-ethnic groups. In terms of discrimination, non-Hispanic White respondents were the least likely to experience or perceive discrimination compared with the other groups. The average level of psychological distress (continuously measured) was also the highest in Korean respondents, followed by Southeast Asian and Hispanic respondents, with White respondents being the least distressed. Black respondents had a higher prevalence of experienced and perceived racial bias than the other minority groups. East Asian respondents reported the highest prevalence of experienced discrimination and the second-highest perceived racial bias among all the minority groups. Among East Asian respondents, Korean respondents reported the highest prevalence of experienced discrimination among all the groups and a prevalence of perceived racial bias similar to that among Chinese and Black respondents. As to socioeconomic resources, Asian respondents were the most advantaged in education and income and similar to White respondents in health insurance and pandemic-time job change experience. Among Asian respondents, East and South Asian respondents were more advantaged in SES than Southeast Asian respondents. Chinese and South Asian respondents had higher household incomes than any other group in this sample.

**Table 1:** Weighted Sample Statistics in the Whole Sample and in Specific Racial/Ethnic Groups

	Psychological	Experienced	Perceived	Has Health	Bachelor’s Degree	Household	Job Change
	Distress (mean/SD)	Discrimination	Discrimination	Insurance	or above	Income^a^	for Worse
Total (N = 2,666)	6.47 (5.59)	8.63%	12.53 (5.30)	91.72%	34.47%	10.05 (4.34)	30.70%
White (N = 509)	5.95 (5.33)	2.73%	10.54 (4.28)	92.90%	37.27%	10.71 (4.16)	27.76%
Black (N = 585)	6.63 (5.73)	19.39%	17.30 (4.88)	92.55%	25.84%	7.91 (4.42)	35.88%
Hispanic (N = 520)	8.01 (6.10)	15.72%	14.72 (5.17)	84.98%	18.13%	8.70 (4.16)	38.69%
Asian (N = 962)	7.11 (5.87)	18.53%	15.85 (5.54)	92.88%	57.11%	10.88 (4.50)	29.36%
East Asian (N = 512)	6.38 (5.40)	22.57%	17.15 (5.14)	95.75%	56.90%	11.17 (4.39)	28.87%
Chinese (N = 306)	5.83 (4.97)	21.74%	17.69 (4.86)	96.84%	59.55%	11.60 (4.31)	25.15%
Japanese (N = 146)	6.42 (5.37)	18.17%	15.83 (5.46)	92.45%	50.33%	10.52 (4.35)	34.32%
Korean (N = 60)	9.55 (6.81)	39.28%	17.56 (5.31)	98.23%	59.00%	9.93 (4.52)	36.11%
South Asian (N = 179)	7.72 (6.11)	10.44%	12.80 (5.25)	92.55%	70.03%	11.66 (4.46)	31.06%
Southeast Asian (N = 215)	8.27 (6.54)	16.16%	15.71 (5.48)	88.95%	43.38%	9.68 (4.53)	29.36%
Other Asian (N = 56)	6.74 (5.30)	19.87%	15.13 (6.14)	84.65%	72.28%	10.63 (4.63)	27.89%

a. Income: 8 = $35,000 to $39,999; 10 = $50,000 to $59,999; 12 = $75,000 to $84,999

Table 2 shows the results from three sequentially nested models of weighted multivariable linear regression of psychological distress. In Model I, when only demographic characteristics were controlled for, Hispanic (coefficient = 1.341; *P* < 0.01), Korean (coefficient = 2.217; *p* < 0.05), Southeast Asian (coefficient = 1.387; *P* < 0.05) groups showed significantly higher levels of psychological distress than non-Hispanic White group. Model II added four SES variables to Model (I) College or above education (coefficient = -0.599; *P* < 0.10), household income (coefficient = -0.151; *P* < 0.001), and having health insurance (coefficient = -1.975; *P* < 0.05) were negatively associated with psychological distress, while experiencing negative job change (coefficient = 1.653; *P* < 0.001) was a positive covariate. In Model II, the coefficient for the Hispanic group was rendered non-significant. This finding suggests that the higher levels of psychological distress among Hispanic respondents are primarily attributable to their lower prevalence of college education, lower household income, higher prevalence of experiencing negative job changes, and higher prevalence of not having health insurance. By contrast, the coefficients of Korean and Southeast Asian ethnicities remained unchanged, indicating that SES cannot explain the higher distress levels among Koreans and Southeast Asians. In addition, the coefficient of the South Asian group became positive and significant in Model (II) This finding implies that SES is a suppressing factor for the South Asian group, in that their higher SES helps mute the manifestation of their psychological distress. The higher levels of psychological distress only surface when SES factors are held constant.

**Table 2:** Coefficients of Weighted Multivariable Liner Regression of Psychological Distress

	Model I	Model II	Model III
**Race/ethnicity**			
White	*Reference*	*Reference*	*Reference*
Black	-0.036	-0.488	-1.598***
	(0.409)	(0.407)	(0.435)
Hispanic	1.341**	0.718	0.013
	(0.483)	(0.480)	(0.477)
East Asian - Chinese	-0.321	0.164	-1.203*
	(0.478)	(0.470)	(0.532)
East Asian - Japanese	0.765	0.693	-0.312
	(0.609)	(0.572)	(0.633)
East Asian - Korean	2.127*	2.361*	0.710
	(1.049)	(1.037)	(1.027)
South Asian	1.104	1.523*	1.051+
	(0.688)	(0.662)	(0.638)
Southeast Asian	1.387*	1.386*	0.460
	(0.640)	(0.640)	(0.657)
Other Asian	-0.146	0.039	-0.910
	(0.808)	(0.754)	(0.740)
Other race	0.229	0.463	-0.512
	(0.835)	(0.804)	(0.810)
**Socioeconomic resources**			
College degree or above		-0.599+	-0.585
		(0.352)	(0.356)
Household income		-0.151***	-0.126**
		(0.044)	(0.043)
Job change for worse		1.653***	1.480***
		(0.365)	(0.364)
Have health insurance		-1.975*	-1.874*
		(0.820)	(0.835)
**Discrimination**			
Experienced discrimination			1.945***
			(0.545)
Perceived discrimination			0.133***
			(0.037)
**Demographics**			
**Age group**			
18–29	*Reference*	*Reference*	*Reference*
30–44	-0.820	-0.610	-0.691
	(0.597)	(0.560)	(0.552)
45–59	-3.077***	-2.557***	-2.647***
	(0.645)	(0.623)	(0.620)
60+	-4.381***	-3.864***	-3.914***
	(0.591)	(0.560)	(0.556)
Male vs. female	-1.071**	-0.979**	-1.101**
	(0.356)	(0.342)	(0.340)
Married vs. not married	-1.024**	-0.533	-0.457
	(0.392)	(0.384)	(0.383)
Number of children	-0.141	-0.126	-0.178
	(0.144)	(0.136)	(0.133)
US-born vs. foreign-born	0.322	0.396	0.354
	(0.519)	(0.535)	(0.527)
**Constant**	9.365***	11.789***	10.142***
	(0.823)	(1.184)	(1.335)

Sample size = 2,666; Exponentiated coefficients; Standard errors in parentheses; + p < 0.10; * p < 0.05; ** p < 0.01; *** p < 0.001

Model III added two discrimination variables, the hypothesized mediators, to Model II. Both experienced discrimination (coefficient = 1.945; *P* < 0.001) and perceived racial bias (coefficient = 0.133; *P* < 0.001) were significantly and positively associated with psychological distress. The coefficients of Korean, South Asian, and Southeast Asian ethnicities became non-significant at the 5% level in Model III, indicating that discrimination played a salient role in contributing to these three Asian groups’ higher levels of psychological distress. Interestingly, the coefficients of Black and Chinese respondents became significant and negative in Model III, whereas they were both positive and non-significant in Model II. This result shows that without discrimination, either experienced or perceived, Black and Chinese respondents would have had less psychological distress than White respondents. In this case, discrimination is a suppressing factor for Black and Chinese respondents’ negative effects on psychological distress compared with White respondents.

Table 3 presents the results of the mediation analysis for the three Asian ethnicities exhibiting positive and significant effects on psychological distress in Model II after socio-demographic factors are controlled. For Korean respondents, the two discrimination variables explained 82% of the total group effect, with experienced discrimination explaining 51% and perceived racial bias explaining 31%. For South Asian respondents, the two discrimination variables explained 34% of the total group effect, with experienced discrimination explaining 11% and perceived racial bias explaining 23%. For Southeast Asian respondents, the two discrimination variables completely explained the total group effect, with experienced discrimination explaining 26% and perceived racial bias explaining 76%.

**Table 3:** Percent of Total Effects of Asian Ethnicities on Psychological Distress Explained by Experienced and Perceived Discrimination

	% Explained by Both Mediators	% Explained by Experienced Discrimination	% Explained by Perceived Discrimination
Korean	81.60%	51.01%	30.61%
South Asian	33.51%	10.51%	23.00%
Southeast Asian	> 100%	26.26%	76.37%

All the mediating effects reported here are significant at the 5% level

## Discussion

To our knowledge, this is the first national study to examine racial-ethnic differences in mental health during the COVID-19 pandemic that included specific Asian ethnicities and explored the psychosocial mechanisms underlying these subgroup differences. Poor mental health was common in all racial-ethnic groups during the pandemic, with the prevalence of moderate psychological distress ranging from 48% among White respondents to 76% among Koreans and those of severe distress ranging from 10% among Chinese to 29% among Southeast Asians. Having accounted for demographic background, among the four major racial-ethnic groups in the US, Hispanic respondents had the highest levels of psychological distress, followed by Asian and then Black respondents, with White respondents exhibiting the lowest levels of distress. Meanwhile, sizeable mental health differences existed among Asian subgroups, where Koreans reported the highest levels of psychological distress and Southeast Asians had the second-highest levels of psychological distress in general. The results from the mediation analysis show that differences in experienced and perceived discrimination contribute to higher levels of psychological distress among Korean, South Asian, and Southeast Asian groups, but not the other racial-ethnic minority groups. For Hispanic respondents, SES factors were the main mechanisms explaining their higher levels of psychological distress.

The finding that Hispanic respondents have been the most distressed racial-ethnic group during the pandemic is consistent with other studies that either also used the K6 scale to measure mental health [12, 36] or focused on other mental problem measures (e.g., depression, suicidal ideation, and substance use) [10, 47]. There seems to be converging evidence that mental health in the COVID-19 era is worst among Hispanic respondents, at least relative to White and Black respondents. These studies did not specifically study Asians in their samples.

In our study, after SES factors were added to the model, the coefficient of the Hispanic group was nearly halved in size and became statistically non-significant. In this sample, the Hispanic respondents were the most socioeconomically disadvantaged among the major racial-ethnic groups, having the lowest percentages in health insurance coverage, college education, and household income and the highest proportion of experiencing a negative job change during the pandemic. These socioeconomic deprivations may transfer to poor mental health via more proximate psychological processes that are detrimental to mental health, such as worrying about job loss, food and housing insecurity, and not being able to obtain needed health care. Indeed, McKnight-Eily et al. documented that a higher percentage of Hispanic than White respondents reported not having enough food or stable housing [10].

Meanwhile, Hispanic communities in the US are also facing intensified discrimination [30], probably due to a backlash in public opinion against immigrants induced by the believed foreign origin of SARS-CoV-2, the coronavirus causing COVID-19, and the role of international travel in the initial spread of the pandemic. In this study, while we found that the higher levels of psychological distress among Hispanic respondents were primarily attributable to their socioeconomic vulnerabilities, slightly more than half of the original Hispanic effect size remained after SES was controlled. The remaining Hispanic effect largely disappeared after the two discrimination variables were further controlled. Although no formal mediation analysis was performed, this finding suggests that discrimination also contributed to worse mental health among Hispanic respondents.

While Asian respondents seemed to be less distressed than Hispanic respondents on average, we found remarkable within-Asian mental health disparities. Among all the racial-ethnic groups examined in this study, Koreans and Southeast Asians had the highest levels of distress and the highest prevalence of moderate and severe distress, whereas Chinese had the lowest level of distress and the lowest prevalence of severe distress. Asian Americans are among the fastest growing and the most ethnically diverse of all major racial-ethnic groups in the US [48, 49]. In this study, we observed a large Korean-Chinese gap in mental health, a pattern consistent with previous evidence that Koreans were more distressed than other Asian groups in the US [50–53]. These findings demonstrate the importance of including Asian Americans in health disparity research and disaggregating the Asian American group by ethnicities to better illuminate mental health disparities by race-ethnicity in the US [54].

After SES was controlled for, three groups exhibited significantly higher levels of psychological distress than White respondents, namely Korean, South Asian, and Southeast Asian respondents, primarily due to the mediating role of experienced discrimination and perceived racial bias. Experienced discrimination taps the respondent’s personal experience of being a victim of direct racial discrimination. Perceived racial bias captures the respondent’s perception of racial bias toward his or her own racial-ethnic group during the COVID-19 pandemic. These views are presumably formed by personally experienced racism and/or vicarious racial discrimination channeled through witnessing or learning about other racial-ethnic group members’ discrimination experiences on news outlets or social media [55]. The effects of the two discrimination measures on psychological distress were both significant and positive and independent of each other. They explained all of the Southeast Asian effect, most of the Korean effect, and about a third of the South Asian effect, after SES indicators were controlled. These findings provide direct evidence that anti-Asian racism and xenophobia, exacerbated during the COVID-19 pandemic [25, 56], is a major culprit for the worsened mental health conditions in Asian American communities.

Between the two discrimination measures, perceived racial bias—tapping vicarious discrimination experiences irrespective of personal experiences of racism—played a more salient role in mediating the Asian ethnic group effects than experienced discrimination for South and Southeast Asians, while for Koreans the opposite was the case. This difference may have been due to East Asians’ higher likelihood of being personally victimized by anti-Asian racism, xenophobia, bigotry, and hatred. Indeed, in our sample, 10% of South Asians and 16% of Southeast Asians reported having personally experienced discrimination, versus 39% of Koreans, 22% of Chinese, and 18% of Japanese reporting this experience. The anti-Asian sentiments and acts that have percolated throughout the US and globally [57] apparently have far-reaching rippling effects on all peoples of Asian descent, including those who are not East Asian and are less likely to be directly attacked or bullied than East Asians.

It is also noteworthy that the Black group effect was not significant when demographic background and SES were controlled, and the effect became *significantly negative* after discrimination was further controlled. This result implied that had Black respondents not differed from White respondents in discrimination, they would have enjoyed better mental health than White respondents. Another study also documented Black respondents’ better mental health than White respondents after adjusting for sociodemographic and health-related characteristics and various pandemic-related stressors [38]. This means racism suppresses the manifestation of mental health advantages among Black respondents.

We observed a similar pattern for the Chinese group. That is, controlling for demographic background and SES, Chinese and White respondents did not differ in psychological distress; yet, after discrimination was further controlled, the mental health premium associated with Chinese group membership relative to White respondents became statistically significant. Our findings on the Chinese are surprising. Among all the communities of color, the Chinese are perhaps the most vulnerable to the heightened anti-Asian racism, xenophobia, and bias since the first confirmed case of COVID-19 in the US on January 19, 2020 due to several reasons. First, the coronavirus causing COVID-19 and setting off a global pandemic was first detected in Wuhan, China, in late 2019. Second, former President Trump’s repeated use of the phrase “Chinese virus” helped lead to a sharp rise in racist anti-Asian and anti-Chinese discourse on the Internet [58]. Third, the recent hate crimes against Chinese can be traced to much deeper historical roots of anti-Chinese sentiment in the US [59] and the rising unfavorable views of China fueled by the strained US-China relationship even before the pandemic era [60]. Data from our study were consistent with these trends. In our sample, the Chinese respondents did report the highest level of perceived racial bias and the second highest percentage of having personally experienced discrimination. Despite these threats, the Chinese respondents also reported lower levels of psychological distress than any other racial-ethnic groups included in this analysis. More research is needed to examine mental health disparities among specific Asian ethnic groups to see if the patterns we observed are replicable. Particularly, we are curious about whether Chinese and Koreans are really sitting at the two ends of the mental health continuum among Asian Americans. If this is confirmed, qualitative research would be helpful to gain an in-depth understanding of the sources of the remarkable resilience in the Chinese community and the unique burdens of the Korean community during a major public health crisis.

The findings from this study have implications for mitigating racial and ethnic disparities in mental health burden. Addressing racism and mitigating its exposure is critically important for improving mental health for racial and ethnic minority groups, especially in the cases of Blacks, Chinese, Koreans, South Asians, and Southeast Asians, as revealed in our study. Peculiar attention needs to be paid to the highest burden of mental health among Korean Americans and its strong association with exposure to racism. This stands in contrast with the predominant role of socioeconomic disadvantages of Hispanics in its higher mental health burden. These findings suggest that mitigating racial and ethnic disparities in mental health requires a tailored approach that is responsive to the specific risk factors in different racial and ethnic groups.

In light of the challenges for addressing systematic racism, developing effective strategies for coping with racism becomes necessary [29]. There is also a need for reducing the stigma associated seeking mental health services for minority groups. Previous studies found that Asian and Hispanic Americans historically perceived less need for mental health services and had lower utilization of the services [61–65]. There was evidence that the lower utilization of mental health services among Asian Americans had more to do with not knowing where to go for treatment than with less perceived need for the treatment [66]. The pronounced sub-group variations within Asian Americans in both discrimination exposure and psychological distress, as revealed in our study, underscore the need for a culturally and ethnically specific approach to promoting access to mental health services among diverse groups of Asian Americans [67, 68].

Several study limitations should be considered when interpreting this study’s messages. First, the cross-sectional design precludes any causal inference from our findings and can only reveal patterns at one point. Second, response bias must have been present as all the measures were self-reported. Third, selection bias was inevitable due to budget constraints due to this web-based survey’s low overall response rate, and the study was conducted only in English and Spanish. Fourth, the sample size of the Korean group was small (N = 60). We performed a power analysis for this study. While this study had sufficient power to examine most of our hypotheses, investigating the mediating effect of perceived racial bias for the Korean group effect needed more power. Longitudinal or repeated cross-sectional surveys, conducted in multiple languages and equipped with substantially larger sample sizes, need to be undertaken in the future to monitor the continuities and changes in mental health disparities by race and ethnicity.

## Conclusions

Based on a nationally representative sample of US adults, this study showed that racial-ethnic minorities suffered from higher levels of psychological distress than Whites. Compared to Whites, Hispanics’ poorer mental health was mainly due to their socioeconomic deficits and, to a lesser extent, racial discrimination and bias. For three Asian ethnic groups, including Koreans, South Asians, and Southeast Asians, discrimination, experienced or perceived, was a critical mechanism underlying their poorer mental health compared to Whites. Among racial-ethnic minorities, Asians, particularly East Asians, reported the highest prevalence of discrimination. A take-home message from this study is that we must stop racially motivated discrimination to mitigate the negative mental health consequences of a pandemic. Purposefully cultivating public awareness of these issues via policies, programs, and cultural interventions is an essential first step toward tackling racial prejudice, reducing health inequity, and improving population health.

## Data Availability

Data can be requested by sending an email inquiry to the corresponding author Dr. Dejun Su (dejun.su@unmc.edu).

## List of Abbreviations

HEAP: Health Ethnicity and Pandemic
K6: The Kessler Distress Scale–6
NORC: National Opinion Research Center
SES: Socioeconomic status
